# Small Practices, Big (QI) Dreams: Customizing Quality Improvement (QI) Efforts for Under-Resourced Primary Care Practices to Improve Diabetes Disparities

**DOI:** 10.2196/23844

**Published:** 2022-03-18

**Authors:** Sahnah Lim, Nadia S Islam

**Affiliations:** 1 Department of Population Health Grossman School of Medicine New York University New York, NY United States

**Keywords:** electronic health record, quality improvement, health equity, clinical practice guidelines, diabetes

## Abstract

Electronic health record quality improvement (QI) initiatives hold great promise in improving adoption of clinical practice guidelines, including those related to diabetes. QI initiatives implemented in under-resourced primary care settings that primarily serve racial/ethnic minority populations have potential to improve quality of care and ultimately improve diabetes disparities. The “Screen at 23” campaign was launched in 2011 to increase screening for prediabetes and diabetes at lower BMI thresholds (ie, 23 kg/m2) for Asian Americans, in line with the new guidelines put forth by the American Diabetes Association. Here, we describe the implementation of a customized electronic health record QI initiative in under-resourced practices that primarily serve low-income South Asian populations in New York City, designed to increase diabetes screening using updated BMI guidelines and in alignment with the “Screen at 23” campaign. The customization involved the implementation of an innovative, semi-manual alternate solution to automated clinical decision support system (CDSS) alerts in order to address the restrictions on customizing CDSS alerts in electronic health record platforms used in small practice settings. We also discuss challenges and strategies with this customized QI effort. Our experience suggests that multisector partnership engagement, user-centered approaches, and informal strategies for relationship building are even more critical in under-resourced, small practice settings. Relatively simple technological solutions can be greatly beneficial in enhancing small practice capacity to engage in larger-scale QI initiatives. Tailored, context-driven approaches for implementation of equity-focused QI initiatives such as the one we describe can increase adoption of clinical practice guidelines, improve diabetes-related outcomes, and improve health disparities among underserved populations.

**International Registered Report Identifier (IRRID):**

RR2-10.1186/s13063-019-3711-y

## Introduction

Clinical practice guidelines inform clinicians about evidence-based medicine with the goal to improve population health. However, adoption of clinical practice guidelines has been poor due to various factors, including lack of awareness of changing guidelines, complexity and volume of guidelines, clinician attitudes, and misalignment of guidelines with clinical workflow [1-3]. Thus, although guidelines provide the content that informs clinical decision-making, they do not provide a roadmap on how to implement these decisions in real-world settings [4]. Moreover, guidelines can unintentionally widen health disparities when barriers to implementation are not explicitly considered [5]. There has been growing interest in translating clinical practice guideline–based quality improvement (QI) initiatives into practice by addressing barriers to adoption [4], in addition to calls for disparities-focused QI initiatives that address barriers to adoption specifically for underserved populations [6].

There has been an increase in clinical practice guideline–based QI using electronic health records (EHRs) [7] such as clinical decision support systems (CDSS) that include a variety of provider-based point-of-care tools including alerts, condition-specific order sets, diagnostic support, and practice-wide reminders [8]. CDSS and other EHR-based health information technology are an effective way of changing clinical practice behavior [9] and subsequently improving health outcomes. These tools are particularly critical for chronic disease management, including cardiovascular diseases and diabetes [10,11]. The Community Preventive Services Task force has recommended the use of CDSS to prevent and manage cardiovascular diseases [12], and the American Diabetes Association further underscored the potential role of CDSS in reducing diabetes disparities [13].

A recent systematic review of guideline-based CDSS QI initiatives found that there are 4 broad dimensions of challenges to their implementation, which include system use, structure, information quality, and system quality [14]. These implementation barriers are exacerbated for under-resourced practices, including federally qualified health centers and small physician- or family-owned community-based practices [6]. Small practices serve a large proportion of low-income immigrants and minorities, especially in urban settings [15]. In New York City (NYC), small practices comprise 40% of primary care providers (PCPs) and serve NYC’s poorest and most racial/ethnically diverse neighborhoods [16]. CDSS QI initiatives often require an infrastructure that is not readily available to small practices and require purchasing of additional software and applications as well as training on these systems to use them accurately [16,17]. Small practices’ lack of access to or suboptimal participation in QI initiatives, therefore, can potentially widen the gap in provision of quality care to health-disparity populations [18-21].

Like many other clinical practice guidelines, the adoption of guidelines put forth by the American Diabetes Association [22] for diagnosing and treating patients with diabetes has been low. Approximately 30 million Americans have diabetes, of which 24% are undiagnosed [23], and there are significant disparities by race/ethnicity [23]. In particular, South Asians have higher diabetes prevalence compared with some other Asian American subgroups as well as other racial/ethnic groups [24,25]. Despite the high and increasing burden of diabetes, an online survey showed that only 53% of clinicians were using diabetes guidelines routinely, with non-guideline users more likely to be practicing in smaller clinics (patient volume <250 a month) [26].

In the 2015 American Diabetes Association guidelines, recommendations were made for lowering the BMI threshold for screening overweight or obese Asian Americans for prediabetes and diabetes from 25 kg/m^2^ to 23 kg/m^2^ [22]. Compared with all other racial/ethnic groups, the prevalence of undiagnosed diabetes is highest for Asian Americans (50%) [27], in large part due to lower screening rates [28]. In response to the American Diabetes Association guidelines and low adoption of racial/ethnic-specific clinical practice guidelines on diabetes, the “Screen at 23” campaign was launched in 2011 to increase awareness and diagnosis of prediabetes and diabetes at this new threshold [29].

To our knowledge, there have been no published studies describing the implementation process of the Screen at 23 campaign, which is critical for providing insight and guidance to health systems seeking to address diabetes disparities serving Asian patient populations. In this paper, we describe the implementation of an EHR QI initiative in under-resourced practices that primarily serve low-income, limited English-proficient South Asian populations in NYC, designed to increase diabetes screening using updated guidelines and in alignment with the Screen at 23 campaign. Smaller EHR platforms serving independent practices often do not have the capability to customize existing CDSS alerts. In response, we developed and implemented a semimanual alternate solution to automated CDSS alerts that incorporate Asian BMI guidelines. We also discuss strategies and challenges with this customized EHR QI effort and implications for improving diabetes and other health disparities in diverse patient populations, with a particular emphasis on deploying informal, community-engaged approaches.

## Implementation of the Customized EHR QI Initiative

### Overview of the DREAM (Diabetes Research, Education, and Action for Minorities) Initiative

The DREAM (Diabetes Research, Education, and Action for Minorities) Initiative is a 5-year randomized controlled trial to support weight loss and glycemic control efforts among South Asian patients receiving care in a network of PCPs in NYC. Details on project study design are described elsewhere [30]. The DREAM Initiative leverages multisectoral partnerships in its implementation, including the Primary Care Information Project (PCIP) at the NYC Department of Health and Mental Hygiene and EHR vendors representing the 2 systems utilized across study sites (MDLand and eClinicalWorks [eCW]). PCIP implements citywide EHR-based QI initiatives by deploying trained practice facilitators to small practices [31]. Their efforts have demonstrated that implementing QI strategies in these settings can effectively improve clinical outcomes, including increased screening (eg, cervical cancer) and disease management (eg, retinal exams, hemoglobin A_1c_ testing) [32-36].

Design and implementation of this initiative were guided by the Chronic Care Model, which identifies the essential elements of a health care system that encourage high-quality chronic disease care and has been widely used in the implementation of diabetes management interventions [37]. Relevant elements of the model for this initiative include delivery system design and clinical information systems, which are addressed by enhancing practice capacity to implement registries of individuals with uncontrolled diabetes. Further guided by PCIP’s best practices [32,33,36] and literature on common challenges faced by under-resourced practices in implementing QI efforts (with emphasis on employing user-centered strategies) [17,36], implementation was conducted in 3 phases. Each phase of the QI initiative, associated activities, and challenges and strategies used to address them are summarized in (Table 1) and briefly described in the following sections.

**Table 1:** Summary of electronic health record (EHR) quality improvement (QI) activities.

QI initiative phases and strategies		Description of activities	Challenges identified and strategies used to address challenges
**Development and customization of registry reports, alerts, and training materials**			
	Develop a customized registry report	Identification of patients at risk of diabetes using revised BMI threshold for Asian Americans Attention to user-friendliness (eg, shortened report run time) with feedback provided from practice facilitators	To monitor fidelity to the QI initiative, it was critical to ensure that registry report generation could be tracked by practices. In developing this feature, a key challenge was identified, namely the differences in report customization process between EHR platforms. In one system (MDLand), reports are customized to allow for tracking of report generation and downloads. In eClinicalWorks (eCW), adding this information to the customized report was not feasible; instead, we provided training on how to download reports to the desktop, which would then require a manual count of the number of downloads.
	Develop semimanual customized alert	Development of a user-friendly workflow to implement the customized alert	Each EHR system required different locations for documenting. After discussion with EHR vendors and PCIP, the team determined that the easiest and fastest way of documentation would be in the chief complaints section for eCW users and internal notes section for MDLand users.
	Develop training manual	Inclusion of the following topics: Review of updated BMI threshold for Asian Americans Systematic documentation of vitals (including BMI) Running customized reports Review of semimanual alternate solution to alerts Inclusion of vendor-specific screenshots of the EHR platform where necessary	The overall goal of the training manual was to provide concise, practical information. With practice facilitator feedback, the training manual underwent multiple rounds of revisions to ensure that only the minimum amount of essential information was communicated. Because of the difference in functionality between the different EHR systems, 2 separate training manuals were developed for each EHR system, including 2 separate suggestions for workflows related to customized alerts in patient charts.
	Deploy customized report	Creation of a temporary username for the practice facilitator EHR vendor deployment of the report for each clinic Practice facilitator/ academic research coordinator testing of the customized report on-site, involving a comparison of the customized report against a random set of individual patient records and noncustomized registry reports	Some practices did not have the technical knowledge to create additional users, and others were hesitant to provide an additional account. In these cases, the practice facilitator/academic research coordinator made an in-person visit to create the user account in the presence of a clinic staff and deleted the account promptly after testing. Testing by the practice facilitator/academic research coordinator required coordination with the clinic during a time that the clinic was not actively using their EHR system during non-business hours. The practice facilitator held a flexible schedule and developed a rapport with clinic staff by offering technical assistance and communicating frequently. If the practice facilitator found errors in the report, the EHR vendors were available to remotely log-in to assess the issue in real time and revise the customized report accordingly.
**Workflow training**			
	Conduct training	Trainings with clinician and clinician staff who are primary users of the EHR Training duration: 1-2 hours, ending with hands-on practice running customized report and implementing the semimanual alert Provision of pdf and hard copies of training manual to trainees	Common to many small practices that experience staff shortage and frequent staff turnover, each staff took on multiple roles. For this reason, training all EHR users was critical. However, coordinating a time for all users at the clinic to be present was logistically difficult. We were able to schedule times during existing team meetings or by engaging a senior-level person at the clinic who was able to effectively guarantee attendance.
**Ongoing technical assistance**			
	Conduct follow-up, in-person technical assistance sessions on a bi-monthly basis	Session duration: approximately 1 hour Review of training manual (if necessary) and customizing of the screening workflow to minimize barriers for implementation Provision of technical assistance on any other EHR issue clinic may be experiencing	Due to high staff turnover, the follow-up session often entailed a new round of training for newly onboarded staff members. The generic workflow suggested during the training session was not manageable to some clinics due to time or workload constraints; this workflow was revised. Rather than following up with the entire list of at-risk patients, the clinic would instead follow-up with 10-15 patients who already had a scheduled appointment in the upcoming month.

### Ethics Approval

The study was approved by the Institutional Review Board of NYU Grossman School of Medicine.

### Phase 1: Development and Customization of Registry Reports, Semiautomated Alerts, and Training Materials

We developed a customized diabetes registry report in collaboration with EHR vendors and PCIP for the purposes of identifying patients at risk for diabetes using the updated BMI criteria for Asian Americans. Reports were customized to ensure user friendliness (eg, selecting essential information from the EHR to minimize the number of columns in the report and minimizing report runtime). Because CDSS alerts are based on clinical practice guidelines for the general population, they could not be customized for specific racial/ethnic groups in the 2 EHR systems at our practices. Instead, we developed a semimanual alternate solution to automated CDSS alerts for patients identified as at-risk. After identifying patients via a customized registry report, eCW users were asked to document the need for screening using the hashtag “#screen@23” in the chief complaints section and MDLand users in the internal notes section. At point of care, the first text that will appear at the top of the progress note is “#screen@23.” We subsequently developed a training manual with topics including updated BMI thresholds for Asian Americans, systematic documentation of vitals, running customized reports, and using the semimanual alert.

### Phase 2: Workflow Training

**Figure 1:**
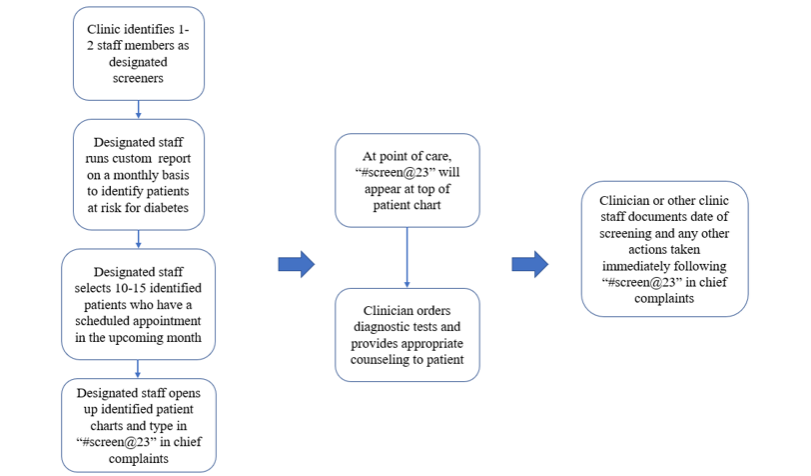
Suggested workflow for identification of at-risk patients and documentation in eClinicalWork's electronic health record platform.

Building upon past successful strategies [11,17,32,38], the practice facilitator and an academic research coordinator conducted an initial 1- to 2-hour training with clinic staff and clinicians. We presented the clinics with a generic suggested workflow (Figure 1) that could be customized to each clinic.

### Phase 3: Ongoing Technical Assistance

Following the initial training, the practice facilitator conducted bimonthly follow-up technical assistance (TA) sessions that included reviewing the manual and re-evaluating the workflow to minimize barriers to screening and documenting. Preliminary feedback indicates that clinics are satisfied with several aspects of the initiative, including the user-friendliness of the reports, the simple workflow for screening and follow-up, and increased awareness on diabetes-related clinical practice guidelines. Some clinics have taken on further customization of workflow; for example, one clinic is now including progress updates for screening at the lower BMI threshold and follow-up of identified patients as a standing agenda item at their monthly team huddles.

The study intervention is conducted in 3 waves across 3 years and 20 primary care practices. To date, we have conducted training with Wave 1 providers (n=7); the practice facilitator collected evaluation data via Salesforce immediately at the end of the in-person training and again during the follow-up TA call approximately 2 months afterwards. All 7 of the Wave 1 providers indicated that the training was very or somewhat useful, they are very or somewhat likely to screen for diabetes using the American Diabetes Association guidelines for a lower BMI for Asians, and they are very or somewhat likely to run the registry reports. At follow-up, 5 of the 7 providers had run the registry report at least once since the training (range: 1-2 times); the 2 providers who did not run the report indicated that they did not remember how to run the reports and were provided with a refresher training.

## Implications for EHR QI Initiatives to Increase Adoption of Clinical Practice Guidelines

The implementation process described here has important implications both for national and local efforts to support the Screen at 23 campaign and more broadly for QI efforts designed to address disparities in diabetes and other health outcomes for minority populations. Although some of the implementation strategies reinforce previous guidance (eg, user-centered designs), other more informal strategies centered around engagement and trust building are more innovative and relevant especially when working with smaller, under-resourced practices who serve minority patients. These implications are summarized in the following sections.

### Multisector Partnership Engagement Is Even More Critical to Success of QI Efforts for Under-Resourced Settings

A critical component of our implementation process was early engagement with partners from a wide range of sectors, including municipal agency–supported practice facilitation services and direct engagement with EHR vendors. PCIP’s practice facilitators have established relationships with small practices, have extensive knowledge about the EHR platforms, and can tailor QI initiatives based on individual practice needs. Programmers at EHR vendors often lack clinical contextual knowledge [32]; by having PCIP’s input during the customized report development, EHR vendors were able to incorporate critical contextual knowledge that would have otherwise been missed. Further, only by bringing both PCIP and EHR vendors to the table together were we able to co-develop an alternate solution to automated CDSS alerts (ie, semimanual, customized solution) that incorporates diabetes screening guidelines specific to Asian Americans. Broader conversations with EHR vendors should be initiated so that seemingly simple customizations (eg, different BMI screening criteria for Asian Americans) can be made to the automated CDSS alerts, which would preclude the need for alternate solutions.

As this and other diabetes-related QI initiatives have demonstrated, leveraging multisectoral partnerships can be a promising implementation strategy [39]. Because not all municipalities have access to a specialized entity like PCIP, it is important to find sustainable ways to support similar efforts, which could include financing strategies to increase the practice facilitator workforce [21]. Without explicit resources toward these efforts in engaging small practices, the disparity in adoption of clinical practice guidelines will widen, with ensuing ramifications on quality of care and health outcomes among immigrant, minority populations [18].

### User Satisfaction and Adoption of QI Initiatives Rely on Implementation of User-Centered Approaches at Every Stage of the Process

As the recent systematic review of CDSS QI initiative highlighted, challenges to implementation primarily center on lack of usability [14]. For this reason, user-centered principles guided our implementation at every stage: The customized report was developed such that only the minimal clinically relevant information was included and took less than 2 minutes to run; the training manual was developed to be user-friendly and practical (eg, step-by-step screenshots); we encouraged a flexible work flow for identification and follow-up of at-risk patients that was manageable for clinic staff members’ workload; and lastly, we developed a semimanual alert for screening that was available at point-of-care but did not significantly disrupt clinician workflow (ie, no additional screen changes or clicks required). This ability to implement user-centered principles depended on meaningful engagement and feedback from key stakeholders, which has been similarly emphasized in other studies [14].

### Informal and Formal Strategies to Develop and Sustain Relationships With Primary Users of the QI Initiative Are Essential for Increasing Trust, Legitimacy, and Ultimately, Adoption

In addition to implementation barriers related to user-friendliness, user attitudes (eg, clinician skepticism about utility of CDSS) can significantly impede adoption [14]. Our previous work with small practices demonstrated the utility of applying community-based participatory research approaches to communication and relationship building to surmount these challenges [17]. Indeed, small practices often do not have the resources for a dedicated informatics staff member or an internal informatics department, which can amplify issues of distrust (especially around sharing patient data with external QI implementers). Accordingly, we sought to develop trust by conducting frequent on-site visits from the practice facilitators and the academic research team, being transparent about the procedures (eg, creating and deleting user accounts in the presence of a clinic staff member), and offering TA at each contact. TA was offered on a wide range of EHR issues and not just those related to our Screen at 23 efforts such as assistance with system updates and submitting tickets for technical support. It was equally important that we engaged not just the clinician in the process but also all staff at the clinic since small practice staff often wear multiple hats to offset the common challenge of staff shortage. Lastly, sustained contact in the form of formal follow-up TA sessions helped with continued communication and increased accountability for adoption of the initiative.

## Conclusion

A common thread underpinning the implementation strategies discussed in this paper is the importance of tailoring to the context of each clinic and using informal strategies to build trust, especially critical in small practice settings due to their relative lack of access to resources. Our body of work engaging small practices [17,21,40] underscores that relatively simple health information technology adjustments can confer great advantage to these under-resourced settings that often provide services to disadvantaged populations. As national trends demonstrate rising diabetes disparities among minority communities [23,41], it is imperative that clinical settings prioritize strategies to improve diabetes-related outcomes among patients. Our experience may provide a road map for tailored, context-driven, and community-engaged approaches for implementation of equity-focused QI initiatives to increase adoption of clinical practice guidelines, improve clinical outcomes related to diabetes, and broadly improve health disparities among underserved populations.

## Abbreviations

CDSS: clinical decision support systems
DREAM: Diabetes, Research, Education, and Action for Minorities
eCW: eClinicalWorks
EHR: electronic health record
NYC: New York City
PCIP: Primary Care Information Project
PCP: primary care provider
QI: quality improvement
TA: technical assistance
